# Gender Differences in Physician Use of Social Media for Professional Advancement

**DOI:** 10.1001/jamanetworkopen.2021.9834

**Published:** 2021-05-13

**Authors:** Nicole C. Woitowich, Vineet M. Arora, Tricia Pendergrast, Michael Gottlieb, N. Seth Trueger, Shikha Jain

**Affiliations:** 1 Department of Medical Social Sciences, Feinberg School of Medicine, Northwestern University, Chicago, Illinois; 2 Department of Medicine, Pritzker School of Medicine, University of Chicago , Chicago, Illinois; 3 Feinberg School of Medicine, Northwestern University, Chicago, Illinois; 4 Department of Emergency Medicine, Rush University Medical Center, Chicago, Illinois; 5 Department of Emergency Medicine, Feinberg School of Medicine, Northwestern University, Chicago, Illinois; 6 *JAMA Network Open*, Chicago, Illinois; 7 Division of Hematology and Oncology, University of Illinois at Chicago, Chicago

## Abstract

This survey study examines gender difference in physician use of social media for professional advancement.

## Introduction

Social media use has been suggested as a method to promote gender equity for women physicians.^1^ This survey study aims to determine gender differences in physicians’ social media use and reported career and professional benefits.

## Methods

This survey study was deemed exempt from review by the Northwestern University institutional review board under category 2(i), which reflects minimal risk to the participant. This study followed the American Association for Public Opinion Research (AAPOR) reporting guideline for survey studies.

A survey to assess physicians’ attitudes and behaviors related to social media (eAppendix in the Supplement) was administered between February and March 2019,^2^ as previously described.^1^ Participants were recruited using a river sampling strategy.^2^ Each of us posted invitations to participate in the survey on the social media platform Twitter using a trackable link and encouraged individuals to share the post within their networks.

We used χ^2^ tests for independence to compare categorical survey responses by gender. Analyses were performed using GraphPad Prism statistical software version 7.0 (GraphPad Software). *P* values were 2-sided, and *P* < .05 was considered statistically significant. Data were analyzed from November 2020 to January 2021.

## Results

Overall, the survey link received 1103 unique clicks and 582 responses (conversion rate, 53%). Of 582 responses, 5 respondents did not describe their gender and were excluded from further analyses, yielding a final sample of 577 respondents. Of the 577 respondents (median [interquartile range] age, 37 [33-44] years), 321 (56%) identified as women and 256 (44%) as men. Most respondents identified as White (425 respondents [74%]), were nontrainee physicians (426 respondents [74%]), and resided in North America (490 respondents [85%]). Most respondents’ primary work was clinical (423 respondents [73%]), and 261 respondents (45%) were academic faculty (Table). Most respondents heard about this study from Twitter (463 respondents [80%]) or Facebook (98 respondents [17%]).

**Table.** Respondent Demographic Characteristics

Characteristic	No. (%)		
	Total (N = 577)	Male (n = 256)	Female (n = 321)
Race			
White	425 (74)	190 (74)	235 (73)
Asian	101 (17)	46 (18)	55 (17)
Black	17 (3)	6 (2)	11 (3)
Other^a^	25 (4)	11 (4)	14 (4)
Prefer not to say	9 (2)	3 (1)	6 (2)
Ethnicity			
Hispanic or Latinx	33 (6)	18 (7)	15 (5)
Not Hispanic or Latinx	544 (94)	238 (93)	306 (95)
Location			
North America	490 (85)	211 (82)	279 (87)
Europe	44 (8)	25 (10)	19 (6)
Asia	25 (4)	10 (4)	15 (5)
Other	18 (3)	10 (4)	8 (2)
Trainee status			
Nontrainee	426 (74)	189 (74)	237 (74)
Resident or fellow	103 (18)	45 (18)	58 (18)
Medical student	33 (6)	16 (6)	17 (5)
Other	15 (3)	6 (2)	9 (3)
Primary work environment			
Clinical care	423 (73)	182 (71)	241 (75)
Education	62 (11)	28 (11)	34 (11)
Administration	31 (5)	21 (8)	10 (3)
Other	61 (11)	25 (10)	36 (11)
Academic faculty			
Yes	261 (45)	120 (47)	141 (44)
No	316 (55)	136 (53)	180 (56)

^a^ Respondents self-described other race/ethnicity.

Both men and women physicians reported using social media to build their professional network (177 men [69%] vs 215 women [67%]; *P* = .58) and agreed that social media use led to increased collaborations with individuals within (178 men [70%] vs 219 women [68%]; *P* = .53) and outside their specialty (152 men [59%] vs 181 women [56%]; *P* = .53) and outside their institution (167 men [65%] vs 202 women [63%]; *P* = .75) (Figure). Men and women physicians also agreed that using social media professionally improved their job satisfaction (145 men [57%] vs 157 women [49%]; *P* = .05).

**Figure.**
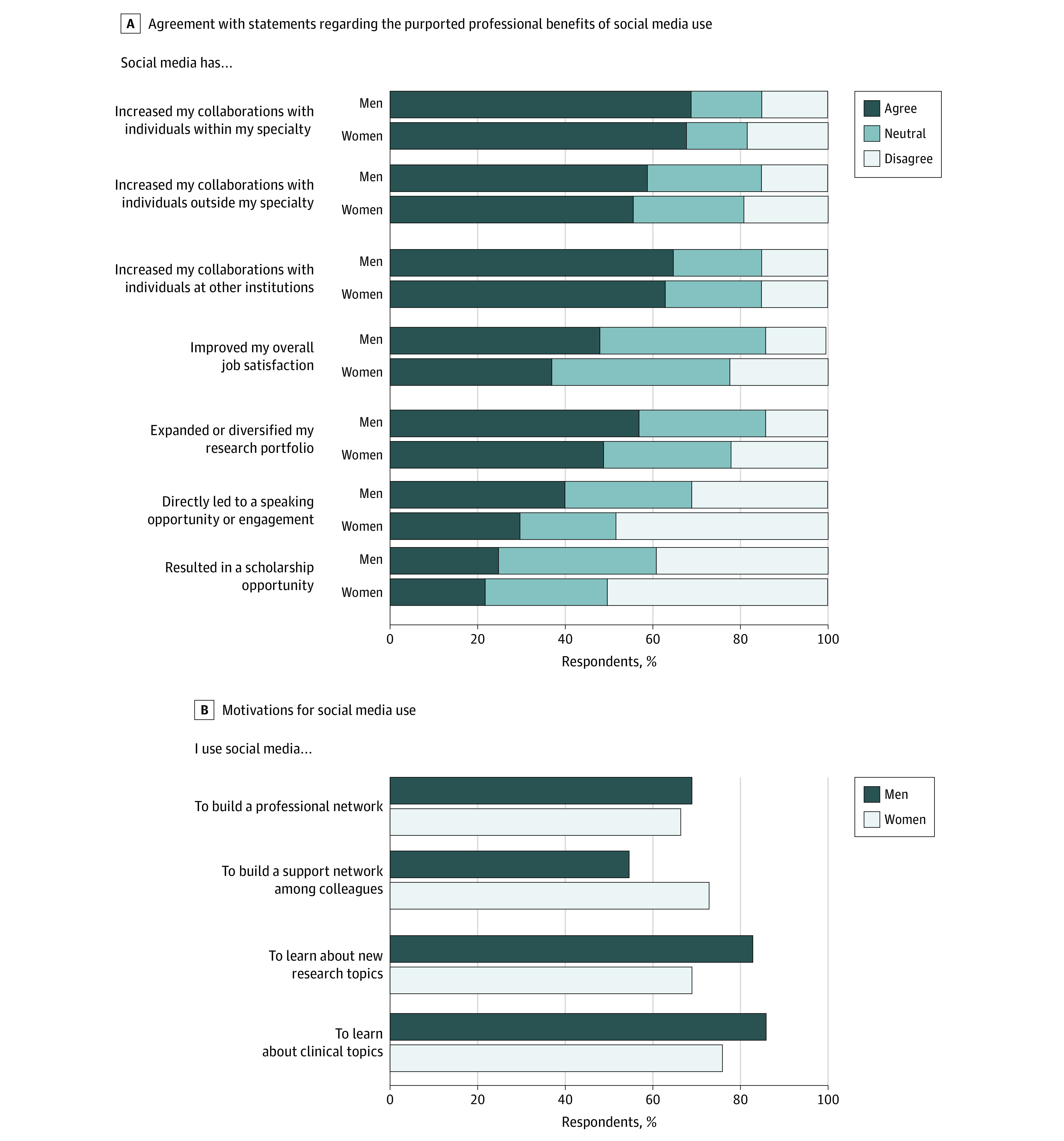
Comparison of Respondents Self-reported Benefits and Use of Social Media by Gender

Compared with men, women physicians were less likely to report that social media use expanded their research portfolio (123 men [48%] vs 117 women [36%]; *P* = .005) or resulted in a speaking engagement (101 men [39%] vs 95 women [30%]; *P* < .001) or scholarship opportunity (63 men [25%] vs 69 women [21%]; *P* = .02). Compared with men, women physicians were more likely to report using social media to build a support network (141 men [55%] vs 233 women [73%]; *P* < .001) (Figure, B) but were less likely to report using it to learn about research (212 men [83%] vs 221 women [68%]; *P* < .001) or clinical topics (219 men [86%] vs 243 women [76%]; *P* = .003).

## Discussion

In this survey study examining physicians’ perceptions of professional benefits of social media use, both men and women felt that social media increased collaboration within and outside their specialties and institutions. However, men physicians were more likely to report certain professional benefits from their social media use, such as invited talks and increased scholarship opportunities. While this study did not assess social media activity or influence, a study from the Association of Medical Colleges^3^ reported that women researchers had fewer followers, retweets, and likes compared with male peers. This may result in reduced visibility of women physicians on social media, despite women explicitly using it to expand their professional network.

This study has some limitations. First, our sample included fewer physicians identifying as Black or other minority race/ethnicities who may face additional disparities,^4^ relative to the physician workforce as a whole.^3^ Second, our survey questions are liable to subjectivity. Third, this study may be subject to self-selection bias to respond to or share the survey.

The findings of this survey study suggest that social media use by women physicians may not improve gender equity. It may be the case that the same biases that lead to fewer opportunities for professional advancement for women persist in the online physician community, hindering women’s professional advancement. Future work to ameliorate this gender disparity is warranted.

## Notes

Supplement. **eAppendix.** Anonymous Social Media Survey
