# Supplementary material for: Gender Differences in Physician Use of Social Media for Professional Advancement
Source: JAMA Netw Open. 2021 May 13;4(5):e219834. doi: 10.1001/jamanetworkopen.2021.9834 (PMC8120326; doi:10.1001/jamanetworkopen.2021.9834)
Supplement: Supplement. — eAppendix. Anonymous Social Media Survey [file jamanetwopen-e219834-s001.pdf]

Supplemental Online Content

Woitowich NC, Arora VM, Pendergrast T, Gottlieb M, Trueger NS, Jain S. Gender differences in physician use of social media for professional advancement. *JAMA Netw Open*. 2021;4(5):e219834. doi:10.1001/jamanetworkopen.2021.9834

eAppendix. Anonymous Social Media Survey

This supplemental material has been provided by the authors to give readers additional information about their work.

© 2021 Woitowich NC et al. *JAMA Network Open*.

Anonymous Social Media Survey

Please complete the survey below.

Thank you!

If you wish to participate, please click "Yes" next to the I Agree text, and you will be taken to the survey.

If you do not wish to participate in this study, please select "No" or select "X" in the corner of your browser.

I agree:	◯ Yes<br>◯ No
What is your age?	______________________________
Where are you from?	◯ United States<br>◯ Puerto Rico<br>◯ Canada<br>◯ Australia<br>◯ United Kingdom<br>◯ Central America<br>◯ South America<br>◯ Africa<br>◯ Asia<br>◯ Mexico<br>◯ Central America<br>◯ Other
Please specify:	______________________________

© 2021 Woitowich NC et al. *JAMA Network Open*.

Which state do you currently live in?	◯ Alabama<br>◯ Alaska<br>◯ Arizona<br>◯ Arkansas<br>◯ California<br>◯ Colorado<br>◯ Connecticut<br>◯ Delaware<br>◯ Florida<br>◯ Georgia<br>◯ Hawaii<br>◯ Idaho<br>◯ Illinois<br>◯ Indiana<br>◯ Iowa<br>◯ Kansas<br>◯ Kentucky<br>◯ Louisiana<br>◯ Maine<br>◯ Maryland<br>◯ Massachusetts<br>◯ Michigan<br>◯ Minnesota<br>◯ Mississippi<br>◯ Missouri<br>◯ Montana<br>◯ Nebraska<br>◯ Nevada<br>◯ New Hampshire<br>◯ New Jersey<br>◯ New Mexico<br>◯ New York<br>◯ North Carolina<br>◯ North Dakota<br>◯ Ohio<br>◯ Oklahoma<br>◯ Oregon<br>◯ Pennsylvania<br>◯ Rhode Island<br>◯ South Carolina<br>◯ South Dakota<br>◯ Tennessee<br>◯ Texas<br>◯ Utah<br>◯ Vermont<br>◯ Virginia<br>◯ Washington<br>◯ West Virginia<br>◯ Wisconsin<br>◯ Wyoming<br>◯ Washington DC
What is your race/ethnicity?	◯ White<br>◯ Black<br>◯ Asian<br>◯ American Indian/Native Alaskan<br>◯ Native Hawaiian<br>◯ Other<br>◯ Unknown<br>◯ I would prefer not to say
Please describe:	______________________________

© 2021 Woitowich NC et al. *JAMA Network Open*.

Do you identify as Hispanic/Latino?	◯ Yes<br>◯ No
How do you identify?	◯ Female<br>◯ Male<br>◯ Non-binary/third gender<br>◯ Prefer to self-describe<br>◯ Prefer not to say
Please specify:	________________________________
Please select your relationship status:	◯ Single<br>◯ Married<br>◯ Partnered<br>◯ Divorced<br>◯ Other
Please describe:	________________________________
What type of degree(s) do you hold?	☐ MD<br>☐ DO<br>☐ MBBS<br>☐ Other international medical doctorate<br>☐ Additional advanced degree
Please specify:	________________________________

© 2021 Woitowich NC et al. *JAMA Network Open*.

Please select the specialty you primarily practice in:

- ◯ Allergy and immunology
- ◯ Anesthesiology
- ◯ Aerospace medicine
- ◯ Cardiology
- ◯ Cardiothoracic surgery
- ◯ Child and adolescent psychiatry and psychotherapy
- ◯ Clinical neurophysiology
- ◯ Colon and Rectal Surgery
- ◯ Critical Care
- ◯ Dermatology
- ◯ Emergency medicine
- ◯ Endocrinology
- ◯ Family Medicine
- ◯ Gastroenterology
- ◯ General practice
- ◯ Geriatrics
- ◯ Obstetrics and gynaecology
- ◯ Health informatics
- ◯ Hospice and palliative medicine
- ◯ Infectious disease
- ◯ Internal medicine
- ◯ Internal medicine subspeciality not listed
- ◯ Interventional radiology
- ◯ Vascular medicine
- ◯ Nephrology
- ◯ Neurology
- ◯ Neurosurgery
- ◯ Nuclear medicine
- ◯ Occupational medicine
- ◯ Ophthalmology
- ◯ Orthodontics
- ◯ Orthopaedics
- ◯ Oral and maxillofacial surgery
- ◯ Otorhinolaryngology
- ◯ Pathology
- ◯ Pediatrics
- ◯ Pediatric cardiology
- ◯ Pediatric critical care
- ◯ Pediatric endocrinology and diabetes
- ◯ Pediatric gastroenterology, hepatology and nutrition
- ◯ Pediatric haematology and oncology
- ◯ Pediatric infectious diseases
- ◯ Pediatric Subspecialty not listed
- ◯ Neonatology
- ◯ Pediatric nephrology
- ◯ Pediatric respiratory medicine
- ◯ Paediatric rheumatology
- ◯ Pediatric surgery
- ◯ Physical medicine and rehabilitation
- ◯ reconstructive and aesthetic surgery
- ◯ Pulmonology
- ◯ Psychiatry
- ◯ Public Health
- ◯ Radiation Oncology
- ◯ Radiology
- ◯ Sports medicine
- ◯ Neuroradiology
- ◯ General surgery
- ◯ Surgical subspecialty not listed
- ◯ Urology
- ◯ Urogynecology
- ◯ Vascular surgery
- ◯ Other
- ◯ Medical Oncology
- ◯ Hematology
- ◯ Hematology/Oncology
- ◯ MedPeds

© 2021 Woitowich NC et al. *JAMA Network Open*.

My specialty is:	______________________
Are you currently a medical student, resident, fellow, or other trainee?	◯ No<br>◯ Yes, I am a Medical Student<br>◯ Yes, I am a Resident<br>◯ Yes, I am a Fellow<br>◯ Yes, I am a Trainee (not listed)
Number of years in practice post-residency completed:	◯ 0-5<br>◯ 5-10<br>◯ 10-15<br>◯ 15-20<br>◯ >20
Please select your primary work environment:	◯ Clinical care<br>◯ Research<br>◯ Education<br>◯ Administration (i.e. division chief, department chair, assistant dean, medical director)<br>◯ Industry<br>◯ Government or non-profit<br>◯ Other
Do you hold an academic faculty position?	◯ Yes<br>◯ No
Please select which applies to you:	◯ Tenure track<br>◯ Non-tenure track<br>◯ I Don't know
What is your current faculty rank?	◯ Assistant Professor<br>◯ Associate Professor<br>◯ Full Professor<br>◯ Clinical Instructor<br>◯ Other
Please specify:	______________________
Do you use any or all of your legal (given) name on social media?	◯ Yes, only on my personal social media accounts<br>◯ Yes, only on my professional social media accounts<br>◯ Yes, on my both personal and professional social media accounts<br>◯ No, I prefer to remain anonymous or use a pseudonym<br>◯ Other
Please specify:	______________________
Do you list your employer/institutional affiliation on your social media accounts?	◯ Yes, my clinical affiliation<br>◯ Yes, my academic affiliation<br>◯ Yes, both my clinical and academic affiliation<br>◯ Yes, something else<br>◯ No, none
Please specify:	______________________

© 2021 Woitowich NC et al. *JAMA Network Open*.

Please indicate the amount of time you spend on social media per day:

	0-30 min	30-60 min	1-3 hrs	3-5 hrs	>5 hrs
Professional Use	○	○	○	○	○
Personal Use	○	○	○	○	○

Why do you use social media (please select all that apply):

- ☐ Building my professional network
- ☐ Building a support network among colleagues
- ☐ Learning about new research
- ☐ Learning about clinical topics
- ☐ Share my work
- ☐ Medical Education (eg creating blogs or podcasts)
- ☐ Other

Please share your other reasons for using social media:

____

Please describe the impact social media has on your...

	Negative impact	Slightly negative impact	No impact	Slightly positive impact	Positive impact
interaction with patients	○	○	○	○	○
interaction with colleages	○	○	○	○	○

Please indicate if you agree with the following statements:

Social media has...

	Strongly Agree	Agree	Neutral	Disagree	Strongly disagree
...expanded or diversified my research portfolio	○	○	○	○	○
...increased my collaboration with individuals in different specialties	○	○	○	○	○
...increased my collaboration with individuals at other institutions	○	○	○	○	○
...influenced my decision to pursue a new or different career trajectory/led to a career change	○	○	○	○	○
...helped me obtain a promotion	○	○	○	○	○
...improved my overall job satisfaction	○	○	○	○	○
...directly led to a speaking opportunity or engagement	○	○	○	○	○
...resulted in a scholarship opportunity	○	○	○	○	○

© 2021 Woitowich NC et al. *JAMA Network Open*.

...increased my collaboration with individuals within my specialty ◯ ◯ ◯ ◯ ◯

I generally think social media is a good thing for me as a medical professional

◯ Strongly Agree
◯ Agree
◯ Agree somewhat
◯ Neutral
◯ Disagree somewhat
◯ Disagree
◯ Strongly disagree

How do you use the following social media platforms?

	Personal Use Only	Professional Use Only	Professional and Personal use	I do not use this
Facebook	◯	◯	◯	◯
Flickr	◯	◯	◯	◯
Instagram	◯	◯	◯	◯
LinkedIn	◯	◯	◯	◯
Snapchat	◯	◯	◯	◯
Tumblr	◯	◯	◯	◯
Twitter	◯	◯	◯	◯
Youtube	◯	◯	◯	◯
Doximity	◯	◯	◯	◯
Blogs	◯	◯	◯	◯
Reddit	◯	◯	◯	◯
Podcasts	◯	◯	◯	◯

Are you a member of social media group specifically for physicians (e.g. facebook groups such as Physician Mom Group, Doctors on Social Media, Dual Physician Families)?

◯ Yes
◯ No
◯ Other
◯ Unsure
◯ Not applicable

Please specify other:

____

Are you a member of a social media group specific to your specialty/subspecialty?

◯ Yes
◯ No
◯ Other
◯ Unsure
◯ Not applicable

Please specify other:

____

Are you a member of a social media group specific to your gender?

◯ Yes
◯ No
◯ Other
◯ Unsure
◯ Not applicable

© 2021 Woitowich NC et al. *JAMA Network Open*.

Please specify: ____________________

How do you share your social media contact information (e.g. blog URL, Twitter handle)?
- ☐ Email signature
- ☐ Business cards
- ☐ Lecture slides
- ☐ Other
- ☐ I don't use social media

Please specify: ____________________

I am concerned my use of social media can have negative impact on my...

	Strongly disagree	Disagree	Neutral	Agree	Strongly agree
...time management	◯	◯	◯	◯	◯
...supervisors' or institutions' perceptions of me and my work	◯	◯	◯	◯	◯

Please note that the following questions contain content regarding sexual harassment.

Have you ever been personally targeted or attacked on social media?
- ◯ Yes
- ◯ No

If you desire, please provide more information about instances of personal targeting on social media: ____________________

Have you ever been sexually harassed on social media?
- ◯ Yes
- ◯ No

If you desire, please provide more information about the instances of harassment: ____________________

The following items are free-response.

Please describe how you think social media has had a positive impact on your overall life: ____________________

Please describe how you think social media has had a positive impact on your career: ____________________

Please describe how you think social media has had a negative impact on your overall life: ____________________

Please describe how you think social media has had a negative impact on your career: ____________________

© 2021 Woitowich NC et al. *JAMA Network Open*.

What do you hope to get out of social media?	__________________________________
Where did you hear about this survey?	☐ Twitter<br>☐ Facebook<br>☐ Instagram<br>☐ LinkedIn<br>☐ Snapchat<br>☐ Doximity<br>☐ Podcasts<br>☐ Blogs<br>☐ Youtube<br>☐ Reddit<br>☐ Other
Please specify:	____________________________

© 2021 Woitowich NC et al. *JAMA Network Open*.
